# Development of a Freeze-Dried, Heat-Stable Influenza Subunit Vaccine Formulation

**DOI:** 10.1371/journal.pone.0164692

**Published:** 2016-11-16

**Authors:** Alexander Flood, Marcus Estrada, David McAdams, Yuhua Ji, Dexiang Chen

**Affiliations:** 1 Devices and Tools Program, PATH, Seattle, Washington, United States of America; 2 Drug Development Program, PATH, Seattle, Washington, United States of America; University of Adelaide, AUSTRALIA

## Abstract

An influenza pandemic remains a major public health concern. A key strategy to prevent a pandemic is to stockpile and pre-position stable influenza vaccine to allow rapid deployment in response to an outbreak. However, most influenza vaccines today are formulated as liquids that are stable only within a temperature range of 2°C to 8°C and require use of a cold chain, making vaccine transportation, distribution, and storage complicated and expensive, particularly for developing countries. To support the National Strategy for Pandemic Influenza preparedness in the United States and internationally, we developed two lead dry formulations of stable H1N1 influenza subunit vaccines using freeze-drying technology. The stable formulations contain an excipient combination of a disaccharide, such as sucrose or trehalose, and glycine, in addition to a surfactant and phosphate buffer. The freeze-dried vaccines were shown to be safe and remained immunogenic in an in vivo study in mice. Moreover, the lead formulations demonstrated no significant loss of activity after 40 months at storage temperatures of 25°C and 37°C. This stability can be particularly attractive as it could eliminate the need to use a cold chain for vaccine deployment and facilitate integration of vaccine distribution with general drug distribution where appropriate. These freeze-dried thermostable influenza subunit vaccines could also reduce the frequency of vaccine stockpile turnover, offering a cost-effective option for pandemic preparedness.

## Introduction

Influenza epidemics reoccur every year and have a significant impact on public health across the globe. Furthermore, the threat of an influenza pandemic remains a major public concern. Our work supports the National Strategy for Pandemic Influenza as it relates to both the United States’ preparedness and the ability to provide assistance internationally to control influenza outbreaks.

A key element in the International Efforts section of the *Implementation Plan for the National Strategy for Pandemic Influenza* is that vaccines “should be stockpiled and pre-positioned for rapid deployment to help ensure that countries affected by an outbreak of pandemic influenza can launch an effective effort to contain the incipient pandemic” [1–3]. A thermostable and cost-effective influenza vaccine would make this goal achievable. Shipping and successfully deploying vaccines in resource-poor regions is greatly facilitated if the products are stable at ambient temperatures.

Currently, there are two types of influenza vaccine on the market: recently developed live attenuated influenza vaccine as a nasal spray [4] and widely used inactivated influenza vaccine formulations consisting of whole inactivated virus, split virus, or subunit antigens. Our work reported involves an H1N1 influenza subunit vaccine. Most inactivated influenza vaccines are administered by intramuscular injection of a liquid formulation.

The main issue associated with liquid influenza vaccine formulations is that they are stable only when kept within a very narrow temperature range of 2°C to 8°C. This requires use of a cold chain, which makes vaccine transportation, distribution, and storage complicated and expensive, particularly for developing countries. Elevated temperatures can lead to thermal degradation of a protein antigen, reducing the potency of the vaccine [5]. Low storage temperatures can result in freezing of vaccine products, leading to damage to the tertiary and secondary structures of the protein and resulting in antigen denaturation [6]. The typical shelf life of an influenza subunit vaccine formulation in a liquid state is 1 year when stored between 2°C and 8°C. Therefore, there is a need to develop a vaccine formulation that can withstand the stress of ambient temperatures and freezing. Such a formulation would make vaccine transportation, distribution, storage, and delivery easier, safer, and independent of the cold chain, and it would ultimately make the vaccine more accessible and less expensive. Furthermore, a stable vaccine with an extended shelf life of at least 2 years would facilitate stockpiling and enable immediate availability of large quantities of vaccine for prompt distribution and would support pandemic preparedness in both high-income and developing countries. In addition, such a vaccine would significantly reduce the frequency of stockpile turnover and associated costs.

One of the viable approaches of obtaining stable biologically active macromolecules is to convert them to a dry solid [7]. In general, proteins are more stable and can be stored at room temperature for longer periods of time in a dry state than in the liquid state [8,9]. Several drying technologies—including spray-drying, foam-drying, and freeze-drying—have been developed to prepare biopharmaceuticals as a dry solid. Among them, freeze-drying, or lyophilization, is widely used in manufacturing pharmaceuticals and biologicals and is often the method of choice for drying vaccine products because of its cost-effectiveness, easy control of process, and manufacturing acceptability [10,11]. Freeze-drying is a two-step process: freezing the materials first and then drying them through the removal of water by sublimation under reduced pressure. The success of a freeze-dried formulation depends on both the process and the formulation. In addition to the stability challenge of formulating biopharmaceuticals, both freeze stress and drying stress may affect the structural integrity and thereby the biologic activity of the proteins.

A wide variety of excipients, such as sugars, amino acids, polymers, surfactants, and salts, are often added to formulations to stabilize proteins by suppressing aggregation and surface absorption or to provide physiological osmolality. It is well known that sugars and saccharides, such as sucrose, trehalose, inulin, and dextran, can not only prevent the aggregation and denaturation of proteins in a liquid formulation but also preserve their structural integrity during freezing, drying, and storage in a solid state [12,13]. The amino acid glycine has been used as a buffering agent as well as a bulking agent for lyophilized products [14].

Here, we report the development of a stable, freeze-dried influenza subunit vaccine to extend product shelf life and enable rapid deployment independent of the cold chain. Specifically, we developed a thermostable, freeze-dried formulation of H1N1 influenza subunit vaccine with a shelf life greater than 3 years not only at controlled room temperature but also at 37°C. We also demonstrated that this formulation is scalable and could be applied to other influenza subunit vaccines, such as H5N1 (not included in this publication).

## Materials and Methods

### Materials

Two lots of influenza monovalent H1N1 vaccine (H1N1 California/07/2009 X179A) were provided by Novartis Pharmaceuticals (East Hanover, NJ). The first lot contained 79 μg hemagglutinin (HA)/mL, and the second lot contained 406 μg HA/mL. Sucrose, glycine, sodium phosphate dibasic, sodium phosphate monobasic (USP or NF) and polysorbate 80 were obtained from J.T.Baker (Center Valley, PA). Trehalose dehydrate (USP/NF) was purchased from Pfanstiehl, Inc. (Waukegan, IL). Arginine, dextran 40, mannitol, and NaCl (reagent or analytical grade) were purchased from Sigma-Aldrich (St. Louis, MO).

### Formulation Preparation

The first bulk vaccine lot was concentrated to 450 μg HA/mL using 10 kDa molecular weight cutoff Amicon ultrafiltration (EMD Millipore, Billerica, MA) and added to formulations containing various commonly used excipients (1-week liquid and Round 1 studies), diluting to a final H1N1 influenza subunit vaccine concentration of 45 μg HA/mL.

The second vaccine lot was concentrated to 1800 μg HA/mL and added to formulations containing various excipients (Round 2 and long-term stability studies), diluting to a final H1N1 influenza subunit vaccine concentration of 90 μg HA/mL. All formulations had the targeted pH of 7.2. The prepared vaccine formulations were sterile filtered with 0.22 μm PVDF syringe filters from Sartorius (Bohemia, NY).

### Freeze-drying

In a typical experiment, 2-mL sterile glass serum vials (West Pharmaceutical Services, Exton, PA) were filled with 0.5 mL of formulated vaccine and placed on the lyophilizer tray, and 13-mm bromobutyl lyophilization stoppers (West Pharmaceutical Services, Exton, PA) were partially inserted into the vials, allowing for venting of water vapor.

Freeze-drying was carried out in a Millrock LD85 freeze dryer (Millrock Technology, Kingston, NY). After vial trays were placed on the lyophilizer shelf, which was set at a shelf temperature of 4°C and held for 2 hours, the shelf temperature was cooled to -45°C and held at this temperature for 3 hours. Subsequently, an annealing step—whereby the shelf temperature is raised above the final freezing temperature to allow efficient crystallization of the crystalline bulking agent, glycine—was performed by holding the temperature at -10°C for 3 hours. Then, the shelf temperature was cooled down to -45°C for 3 hours. The temperature ramping rate was 0.408°C/minute during thermal cycling. Primary drying was performed at -25°C for 36 hours and the secondary drying at 30°C for 10 hours. The chamber vacuum pressure was set at 60 mTorr, and the temperature ramping rate was 0.3°C/minute during drying. (Note: for Round 1, the primary drying was performed at -45°C for 24 hours at a vacuum pressure of 100 mTorr). After the completion of freeze-drying, the vials were filled with dry nitrogen and stoppered.

### Single Radial Immunodiffusion Assay

A single radial immunodiffusion (SRID) assay was used to determine the antigenic activity of the H1N1 subunit vaccine either in a liquid formulation or in a dry solid formulation immediately after the formulation preparation process and after storage under study conditions. Gels were prepared by dissolving 1% agarose in phosphate-buffered saline (PBS), adding 10–20 μL/mL of influenza Anti-A/California/7/2009 (H1N1) HA serum (National Institute for Biological Standards and Control [NIBSC], UK), and dispensing on a plastic backing. Wells were punched in a slab of the solidified agarose gel. A dilution series of the reference standard influenza antigen A/California/7/09 (H1N1) (NIBSC, UK) at four concentrations from 50 to 12.5 μg/mL, and the test sample at two concentrations in the linear range of the standard, were prepared in PBS containing 1% of Zwittergent 3–14 (EMD Millipore). After the addition of 20 μL of antigen sample to the well, the antigen diffused into the agarose, resulting in an antigen–antibody reaction causing a zone of precipitation around the well. After a 24-hour diffusion period at room temperature in a humidified chamber, the gel was washed to remove unbound antibody, dried, stained with Coomassie Blue dye (0.1% in a mixture containing 45% ethanol and 10% acetic acid), and then destained. Images of precipitation zones were recorded on an AlphaImager (ProteinSimple, San Jose, CA), and the diameters of the rings were measured using ImageJ software [15]. The ring sizes of both the reference standard and test sample were compared, and HA content in the test sample was determined against the reference standards in units of μg/mL. Triplicate and duplicate measurements were made for each test sample and reference standard analysis, respectively.

### Moisture Content Analysis

Water in a freeze-dried vaccine vial was extracted with anhydrous methanol. The obtained extract was injected into a Mettler Toledo (Columbus, OH) C20 Karl Fischer coulometric titrator to determine the amount of water present in the sample. The water content in the dried powder was calculated by comparing the amount of extracted water to the weight of the lyophilized vaccine formulation.

### In Vivo Immunogenicity Study

The immunogenicity study was conducted at Tulane University School of Medicine, which served as the study service provider. The animal experiments were performed in strict accordance with institutional and national guidelines. The Tulane University Policy of Humane Experimental Endpoints was reviewed by PATH and applied to all animals in the study. Female BALB/c mice (17 to 21 g body weight) were obtained from Charles River Laboratories (Wilmington, MA) and used for the study. Each study group consisted of 10 mice. At weeks 0 and 4, mice were anesthetized via intraperitoneal injection of ketamine (100mg/kg)/xylazine (10mg/kg) and then injected intramuscularly in the caudal thigh muscle (hind leg) with 100 μL of either reconstituted H1N1 subunit vaccine diluted to 0.1 μg/mL and 1.0 μg/mL in phosphate-buffered saline containing 0.05% polysorbate 80, or liquid vaccine directly diluted from bulk vaccine in phosphate-buffered saline containing 0.05% polysorbate 80. Blood was collected via retro-orbital collection on all animals prior to each vaccination (weeks 0 and 4) and at 4 weeks after the final vaccination (week 8). Serum was collected and stored at -20°C until a hemagglutination-inhibition (HAI) assay was performed.

Prior to assay performance, all serum samples were treated with Receptor Destroying Enzyme (RDE) (Accurate Chemical & Scientific Corporation, Westbury, NY) before use in HAI assay. RDE in PBS was added (3:1) to serum and incubated overnight in a 37°C water bath and then at 56°C for 30 minutes to inactivate residual RDE activity. Serum samples were serially diluted twofold in PBS and transferred in duplicate to a V-bottom, 96-well tissue culture plate. Each well was filled with 25 μL of serum sample and 25 μL PBS with the exception of the first wells in each dilution series, which were filled with 50 μL of serum with no PBS. Control wells on each plate contained no serum sample but contained a positive control (ferret hyperimmune serum). After dilution, influenza A/Cal/07/2009 antigen was added to each well (4 HA Units/25 μL, standardized each assay day), and serum–virus mixture was incubated at room temperature for 30 minutes. Finally, a suspension of 0.5% turkey red blood cells (Lampire Biological Laboratories, Pipersville, PA) in PBS was added to each well, and hemagglutination was allowed to proceed for up to 30 minutes at room temperature. The reciprocal of highest serum dilution demonstrating inhibition of hemagglutination was scored as the HAI titer.

### X-ray Powder Diffraction

X-ray powder diffraction (XRPD) patterns of freeze-dried powders were determined at Triclinic Labs (Lafayette, IN) under low-humidity conditions. The powder sample was mounted by sandwiching the loose powder between two Etnom isolation films inside an enclosed glove box flushed with dry nitrogen. A double Etnom barrier isolated the sample from environmental humidity during data collection. Each XRPD pattern was determined using a multiple-frame technique (4 loops) on a focusing transmission X-ray system. Each frame (loop) took about 20 minutes to complete, and the four frames represented about 80 minutes of active data collection time.To determine the amount of crystalline material present in the lead formulation, a full-pattern profile fitting was performed using lyophilized formulations containing sucrose only and glycine only as noncrystalline and crystalline reference patterns, respectively. Crystallinity was calculated using Excel solver software. The resulting model fits to the lead formulation sample data, and the crystalline-to-noncrystalline ratios were estimated.

### Statistical analysis

The statistical significance of differences in the stability results of the lead formulations was evaluated by comparing slopes of linear regression lines using Graphpad Prism 6 (La Jolla, CA). A p-value < 0.05 was considered significant. For initial formulation screening experiments, statistical analysis was not performed due to limited sample size and data points. For the in vivo immunogeneicity study in mice, statistical comparisons of data were carried out using the t-test of Prism software. A p-value < 0.05 was considered to be significant.

## Results

### Formulation Development

In the development of freeze-dried vaccines, the choice of excipients is of major importance, with each excipient performing one or more critical functions. As the first step in developing a stable freeze-dried influenza vaccine formulation, six formulations with various commonly used carbohydrates (sucrose, trehalose, mannitol, dextran 40) and amino acids (glycine, arginine) as stabilizers or bulking agents were prepared, and the HA antigen stability in the liquid formulations was evaluated at 4°C, 37°C, and 45°C for up to 7 days to quickly eliminate any stability-reducing excipient. We also assessed the effect on formulation stability of high (140 mM) and low (14 mM) levels of sodium chloride as a tonicity modifier. The concentrated bulk vaccine was diluted to 45 μg HA/mL as the targeted concentration, and all formulations contained 20 mM of sodium phosphate as a pH buffer and 0.09% polysorbate 80 as surfactant. Table 1 shows the composition of the formulations.

**Table 1:** Excipient Content in Formulations Prepared for Liquid Stability Screening.

Formulation	Sucrose	Trehalose	Mannitol	Glycine	Dextran 40	Arginine	NaH_2_PO_4_	NaCl
	Final Concentration (% w/w)						mM	
F1	5						20	14
F2		5					20	14
F3			5				20	14
F4				5			20	14
F5					5		20	14
F6						5	20	14
F7							20	14
F8							20	140

All formulations contained 0.09% polysorbate 80, which was already present in the bulk vaccine.

**Fig 1:**
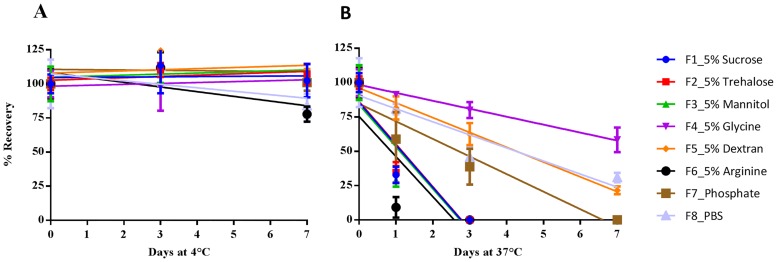
Stability of Subunit Vaccine in Liquid Formulations at 4°C (left) and 37°C (right). Formulations were prepared with concentrated bulk H1N1 subunit vaccine (H1N1 California/07/2009 X179A). In addition to the stabilizers shown in the figure, all formulations contain 45 μg HA/mL, 20 mM phosphate buffer, 0.09% polysorbate 80 and 14 mM NaCl (F8 was in PBS which contained 140 mM NaCl). The formulations were stored at 4°C (A, left) and 37°C (B, right), and their HA titers were measured by SRID. Formulations were tested for up to 7 days. For screening, one dilution for each formulation was tested by SRID in triplicate. The data were presented as percentage recovery relative to the mean HA titer after formulation preparation (time 0) ± standard deviation of each triplicate. Abbreviations: SRID, single radial immunodiffusion; HA, hemagglutinin; PBS, phosphate buffer saline.

The liquid stability of these formulations at 4°C and 37°C was monitored by measuring HA content using SRID at day 0, 1, 3, and 7 (Fig 1).

The formulations containing glycine (F4) or dextran (F5) showed the greatest liquid stability. In fact, only these two formulations showed reasonable stability at a temperature of 45°C on day 1. Formulation F8, with a high concentration of NaCl (140 mM), was more stable than F7, which had one-tenth as much sodium chloride, indicating that reduced ion strength decreased the liquid formulation stability. Furthermore, 5% sucrose (F1) or trehalose (F2) did not sufficiently compensate for reduced ionic strength, in comparison with F8. In this experiment, the stabilizing effect of sucrose, trehalose, and mannitol on the formulations in liquid form was not obvious at 37°C, although these formulations were stable at 4°C. Because the formulation containing arginine showed the lowest stability at both 4°C and 37°C, arginine was excluded from subsequent experiments.

The second step was to evaluate the suitability of the freeze-drying process for the subunit vaccine and formulation stability in solid state. The screening of freeze-dried formulations was performed over two rounds. Table 2 shows the composition of tested formulations.

**Table 2:** Composition of Formulations Used in Screening for Freeze-dried H1N1 Subunit Vaccine.

Formulation		Final Concentration (% w/v)				Final Concentration (mM)		
	ID	Sucrose	Trehalose	Mannitol	Glycine	Dextran 40	Phosphate	Histidine
**Round 1**	Lyo1-F1	4					20	
	Lyo1-F2		4				20	
	Lyo1-F3			3			20	
	Lyo1-F4				3		20	
	Lyo1-F5	1		3			20	
	Lyo1-F6	1			3		20	
	Lyo1-F7	3				1	20	
**Round 2**	Lyo2-F1	4					20	
	Lyo2-F2	1			3		20	
	Lyo2-F3	3				1	20	
	Lyo2-F4		4				20	
	Lyo2-F5		1		3		20	
	Lyo2-F6		3			1	20	
	Lyo2-F7		4					20
	Lyo2-F8		1		3			20
	Lyo2-F9		3			1		20

The targeted final HA concentration was 45 μg/mL in Round 1 and 90 μg/mL in Round 2. The Round 1 formulations contained 14 mM of NaCl and 0.09% of polysorbate 80, while Round 2 formulations contained 7 mM of NaCl and 0.05% of polysorbate 80. The targeted pH was 7.2.

The design of these formulations was largely based on the properties of each excipient and the potential functions of each during the freeze-drying process and dried powder storage [16,17]. The disaccharides sucrose and trehalose were chosen because of their well-known stabilizing effect by forming a sugar-glass matrix during freeze-drying [18,19,20], while the polymer dextran was included to add plasticity to the formulation [21]. Glycine and mannitol are widely used bulking agents that can reduce the duration of the lyophilization cycle and yield dried product with desirable lyophilized cake characteristics [22,23].

**Fig 2:**
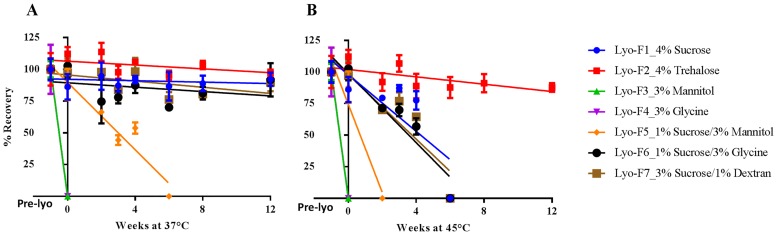
Stability of Freeze-dried Influenza Vaccine Formulations (Round 1) at 37°C (A) and 45°C (B). Formulations were prepared with concentrated bulk H1N1 subunit vaccine (H1N1 California/07/2009 X179A). In addition to the stabilizers shown in the figure, all formulations contain 45 μg HA/mL, 20 mM phosphate buffer, 0.09% polysorbate 80 and 14 mM NaCl. The formulations were freeze-dried as described in Materials and Methods and stored at 37°C (A, left) and 45°C (B, right). The HA titers were measured by SRID up to three months. For screening, one dilution for each formulation was tested by SRID in triplicate. The data were presented as percentage recovery relative to the mean HA titer after formulation preparation but before freeze drying (Pre-lyo) ± standard deviation of each triplicate. Abbreviations: SRID, single radial immunodiffusion; HA, hemagglutinin.

The stability of freeze-dried solid from Round 1 was monitored immediately after freeze-drying (time 0) and after 3 months of storage at three temperatures of 4°C, 37°C, and 45°C (Fig 2). The stability of freeze-dried solid from Round 2 was monitored after freeze-drying (time 0) and after 2 months of storage at three temperatures of 4°C, 37°C, and 48°C.

The antigenic activity of formulations in liquid, prior to freeze-drying, was also determined to monitor the potency loss during processing. These data are shown as before week 0 in Fig 2. The data from Round 1 indicates that the presence of 1% to 4% disaccharide (either sucrose or trehalose) adequately protected the subunit vaccine from damage. The formulation with 4% trehalose (Lyo1-F2) was the best performer, with no significant loss in HA titers during storage for up to 3 months at 45°C. Although a collapse of cake structure and subsequent loss in HA titers of the formulation with 4% sucrose (Lyo1-F1) was observed after 1 month at 45°C, this formulation was stable for up to 3 months at 37°C. The subunit vaccine in the formulations containing mannitol (Lyo1-F3) or glycine only (Lyo1-F4) showed a complete loss of potency after lyophilization. However, the combination of 1% sucrose with mannitol or glycine (Lyo1-F5 and Lyo1-F6) reduced process loss and improved product stability. Lyo1-F6 (1% sucrose and 3% glycine) was more stable than Lyo1-F5 (1% sucrose and 3% mannitol), suggesting that glycine provides better stability than mannitol for freeze-dried H1N1 subunit vaccine. All other formulations exhibited minimal process loss and had no loss in HA titer at 4°C for up to 3 months.

**Fig 3:**
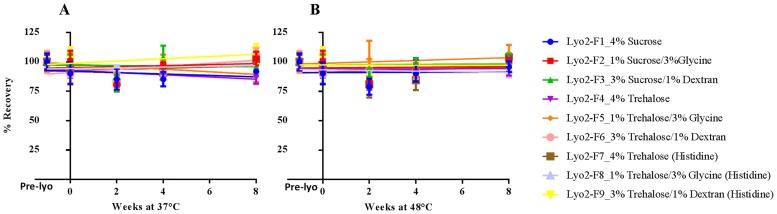
Stability of Freeze-dried influenza vaccine Formulations (Round 2) at 37°C (A) and 48°C (B). Formulations were prepared with concentrated bulk H1N1 subunit vaccine (H1N1 California/07/2009 X179A). In addition to the stabilizers shown in the figure, all formulations contain 45 μg HA/mL, 20 mM phosphate buffer, 0.09% polysorbate 80 and 14 mM NaCl. The formulations were freeze-dried as described in Materials and Methods and stored at 37°C (A, left) and 48°C (B, right). The HA titers were measured by SRID up to two months. One dilution for each formulation was tested by SRID in triplicate. The data were presented as percentage recovery relative to the mean HA titer after formulation preparation and before freeze-drying (Pre-Lyo) ± standard deviation of each triplicate. Abbreviations: SRID, single radial immunodiffusion; HA, hemagglutinin.

The Round 1 results led to further formulation design, with a focus on investigating the disaccharides–glycine-based combination excipient system in Round 2, while keeping the disaccharides-only system as a control. Because of the excellent stabilizing effect of trehalose observed in Round 1, the combination of trehalose with glycine and dextran was included in Round 2 (see Table 2). We also investigated the possibility of an alternative buffer system by replacing sodium phosphate with histidine. In Round 2, we used a new lot of bulk vaccine and we optimized the freeze-drying process by raising the primary drying temperature from -45°C to -25°C. In addition, we shortened the accelerated stability study time from 3 to 2 months but increased the high study temperature from 45°C to 48°C. Fig 3 summarizes the 2-month stability results of Round 2.

All nine formulations from Round 2 remained stable even after 2 months at 48°C, including the sucrose-only formulation, which failed by 6 weeks at 45°C in Round 1. In addition, three formulations (Lyo2-F7, Lyo2-F8, and Lyo2-F9) with histidine as an alternate buffer were also stable. No lyophilized cake collapse was observed for formulations in Round 2 during the accelerated aging studies. Recovery of HA activity after the freeze-drying process was 90–100% in both rounds.

**Fig 4:**
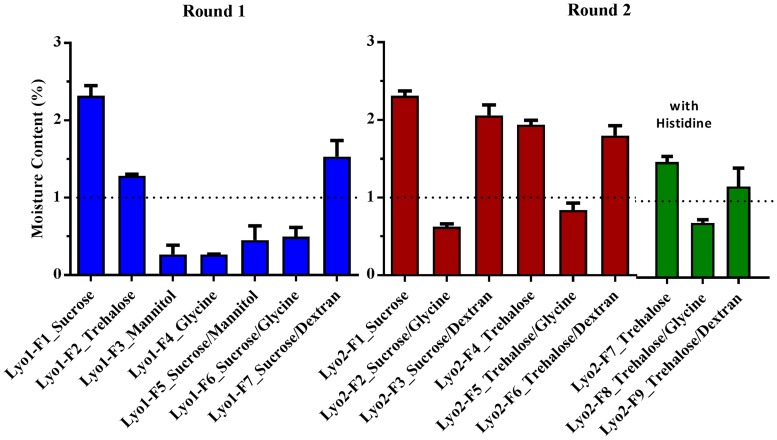
Moisture Contents of all Formulations in Rounds 1 and 2. Water in freeze-dried formulations was extracted with anhydrous methanol and then determined by Karl Fischer coulometry. The percentage of water content in the dried powder (w/w) was calculated by comparing the amount of extracted water to the weight of the lyophilized vaccine formulation. Three vials were measured for each formulation. The data were represented as the mean of the three samples ± standard deviation.

In both rounds, moisture content was lower for formulations containing a bulking agent, either mannitol or glycine, than for formulations with disaccharides only (Fig 4). In Round 2, the moisture content of formulations with sucrose–glycine-based excipients and trehalose–glycine-based excipients was less than 1% H_2_O (w/w), which was lower than that for formulations with sucrose/trehalose–dextran-based excipients. Overall, the moisture content was less than 2.5% H_2_O (w/w) for all formulations.

Considering the stability of the formulation and the cost and function of each excipient, we finally selected two formulations containing both a disaccharide (sucrose or trehalose) at 1% and glycine at 3% as lead formulations for long-term stability and in vivo immunogenicity evaluation.

### Long-Term Stability of Lead Formulations

We studied the long-term stability of the two lead formulations (detailed in Table 3) when stored at 4°C, 25°C, and 37°C. Initially planned for 24 months, the study was extended to 40 months to accurately observe the rate of degradation and predict the shelf life.

**Table 3:** Composition of Selected Freeze-dried Lead Formulations for Long-term Stability Study.

Composition	Lead 1	Lead 2
Sucrose (w/v)	1%	-
Trehalose (w/v)	-	1%
Glycine (w/v)	3%	3%
Sodium phosphate (mM)	20	20
Polysorbate 80^1^ (w/v)	0.05%	0.05%
Hemagglutinin (μg/mL)	90	90
pH	7.2	7.2

^1^ Polysorbate 80 was from bulk vaccine and no additional amount was added.

**Fig 5:**
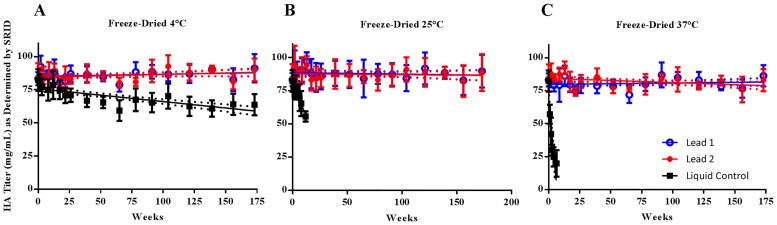
Long-term Stability of Freeze-dried Lead Formulations of H1N1 Subunit Vaccine Stored at 4°C, 25°C, and 37°C. Two freeze-dried lead formulations were prepared with concentrated bulk H1N1 subunit vaccine (H1N1 California/07/2009 X179A) in phosphate buffer and additional excipients listed in Table 3. The liquid formulation obtained from dilution of bulk vaccine with PBS was used as the control. The stability at 4°C (A), 25°C (B), and 37°C (C) was monitored up to 40 months. For each formulation, three vials were reconstituted and combined. Two dilutions were prepared within the linear range of the standard curve and tested by SRID in triplicate. The data represented the mean HA titer ± standard deviation of six measurements.

Formulation Lead 1 exhibited no loss in HA content after 40 months of storage at all storage temperatures. A decrease in HA titer was observed over time for formulation Lead 2 stored at 37°C, but the loss of HA titer was less than 20% and thus the data were within the acceptable range for shelf life determination (Fig 5). Therefore, the stability of both lead formulations demonstrated statistically significant improvement at all storage conditions, compared to the liquid control stored at 4°C (p<0.0001 for Lead 1 at 4°C, 25°C and 37°C; p<0.0001 for Lead 2 at 4°C, 25°C and p = 0.0003 at 37°C). In addition, two lead formulations showed comparable stability (p>0.5 at all conditions).

**Fig 6:**
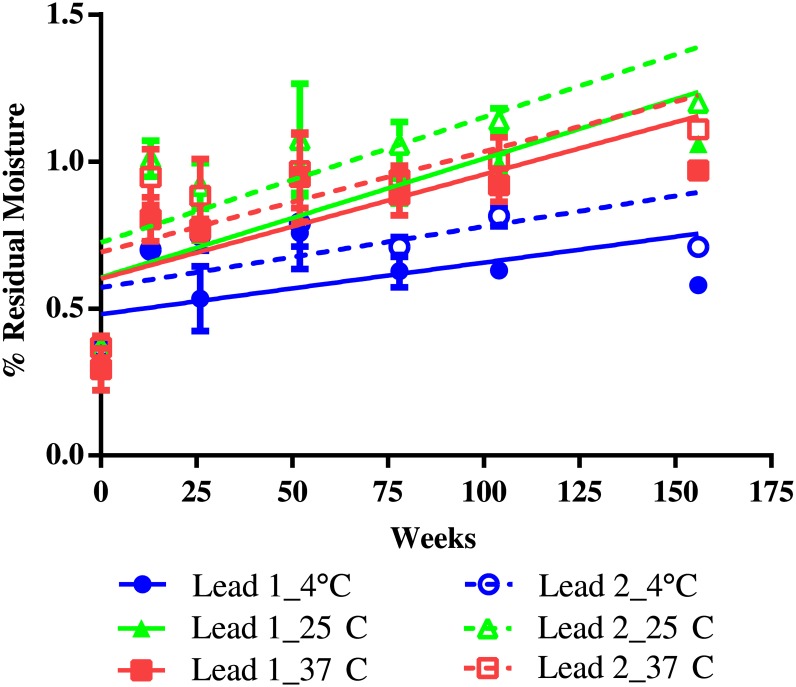
Residual Moisture Content of Freeze-dried Lead Formulations of H1N1 Subunit Vaccine Stored at 4°C, 25°C, and 37°C. The moisture content in stability samples of two freeze-dried lead formulations, stored at 4°C, 25°C, and 37°C, was monitored for three years by using Karl Fischer coulometry. The percentage of water content in the dried powder (w/w) was calculated by comparing the amount of extracted water to the weight of the lyophilized vaccine formulation. Three vials were measured for each formulation at each time point. The data was represented as the mean of the three samples ± standard deviation. Due to the limited number of sample vials, only two vials at week 104 and one vial at week 156 were tested. For the sample stored at 4°C, only one vial was tested at week 12.

Moisture content was monitored up to 36 months (156 weeks). It was initially less than 0.5% H_2_O (w/w) in both formulations but increased to 0.7–1.0% H_2_O (w/w) after 3 months (13 weeks), with slightly higher content at higher temperatures. It remained the same through the 36-month storage period (Fig 6). Visual inspection found no change in physical form after 40 months of storage.

The overall results clearly indicate a minimum of 3 years of stability of both lead formulations under storage temperatures of 4°C, 25°C, and 37°C.

### Immunological Response in Mice

The immunogenicity of freeze-dried influenza H1N1 subunit vaccine powder was evaluated in mice. The study included three groups. For groups 1 and 2, the subunit vaccine powder of freeze-dried formulation Lead 1 and Lead 2 was reconstituted and diluted to 0.1 μg/mL and 1.0 μg/mL in phosphate-buffered saline containing 0.05% polysorbate 80, respectively, while subunit vaccine in PBS was used as a positive control for group 3. The mice received 0.01 μg and 0.1 μg intramuscular injections of the study formulations at weeks 0 and 4, and the serum HAI titer from each animal was determined at weeks 0, 4, and 8 (4 weeks after the final vaccination) and expressed as the reciprocal of the highest dilution demonstrating HA inhibiton. A HAI titer of 1:40 or a four-fold increase in HAI titer induced by vaccine formulation over preimmunization is considered as acceptable efficacy in both non-clinical and clinical settings.

**Fig 7:**
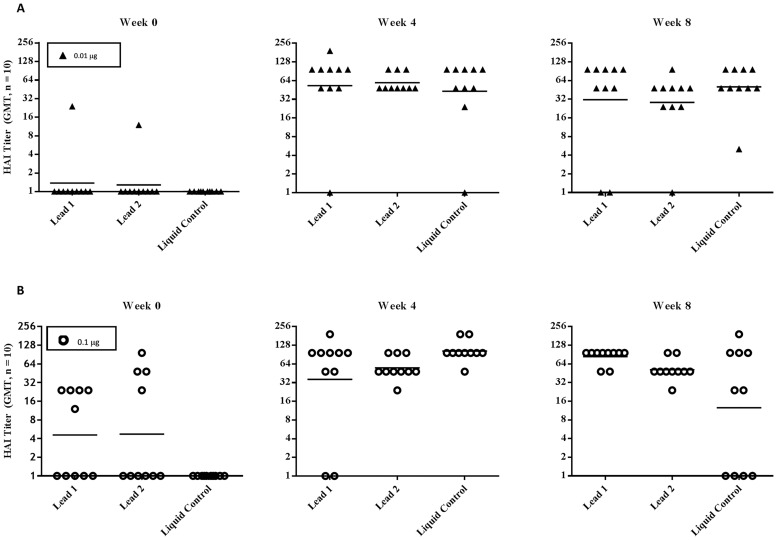
HAI Titers in Mice Immunized with H1N1 (A/Cal/07/2009) Influenza Subunit Vaccine. Each study group consisted of 10 mice. Animals were immunized intramuscularly with 100 μL of either reconstituted freeze-dried H1N1 subunit vaccine or liquid vaccine diluted to 0.1 μg/mL (upper panel: A) and 1.0 μg/mL (lower panel: B) and blood samples were collected prior to vaccination (weeks 0 and 4) and 4 weeks after the final vaccination (week 8). HAI tiers in serum samples against influenza A/Cal/07/2009 were measured in duplicate, at week 0 (left, pre-immunization), week 4 (middle) and week 8 (right), and were expressed as the reciprocal of the highest dilution demonstrating HA inhibition. Data of individual mice are shown. Geometric mean titer of each group is shown by a horizontal bar. HAI titers induced by immunization at week 4 and week 8 were significantly different from HAI titers at baseline (week 0), (p≤0.002 for groups immunized with 0.01 μg and p<0.05 for groups immunized with 0.1 μg.

Fig 7 summarizes the results. At the 0.01 μg dose, HAI titers at week 4 and week 8 were increased more than 4-fold compared to week 0 (pre-immunization) for all arms (p≤0.002). In addition, there was no significant difference in HAI titers between freeze-dried formulation (either Lead 1 or Lead 2) and the liquid control (p>0.19). At the high dose of 0.1 μg, HAI titers after immunization (week 4 and week 8) were significantly increased from the baseline titers at week 0 (p<0.05) for all three groups. HAI titers induced by Lead 1 at week 4 and week 8, and by Lead 2 at week 8, were similar to those of the liquid control (p>0.12), although background HAI titers were observed at week 0 in the groups of freeze-dried formulations, in particular in the group of Lead 2. In this experiment, the second immunization at week 4 failed to show a boost effect. The observation of a decline in HAI titer after the second immunization in the liquid control group was unexpected, and the number of animals per grould should be increased for future study to minimize the variation.

Overall, both freeze-dried lead formulations elicited comparable efficacy to the liquid control. The data confirmed that immunogenicity was not altered in the freeze-dried subunit vaccine formulations. Furthermore, no adverse events were observed for any mice throughout the in-life study period. Weight changes were consistent with control mice that were untreated and cohoused with study animals (data not reported here).

### X-ray Crystallography

**Fig 8:**
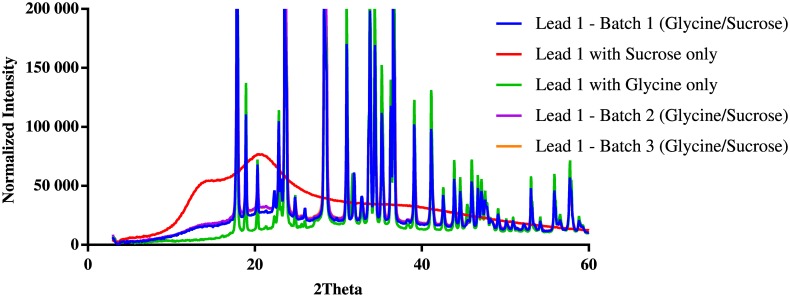
Overlay of X-ray Powder Diffractograms of Freeze-dried Powder. X-ray powder diffraction (XRPD) patterns of freeze-dried powders of three batches of Lead 1 and Lead 1 controls with sucrose only and glycine only were determined with Rigaku SmartLab system at Triclinic Labs (Lafayette, IN) under low-humidity conditions, as described in Materials and Methods.

To understand whether the outstanding stability of the two lead formulations was attributable to the amorphous sugar-glass matrix in the powder mixture with a disaccharide–glycine-based excipient, we obtained the x-ray diffraction (XRD) patterns of three batches of H1N1 subunit vaccine powder of freeze-dried formulation Lead 1. For comparison, we also determined the XRD patterns of freeze-dried formulations containing glycine only and sucrose only. The XRD data analysis (Fig 8) indicates that the freeze-dried powder of formulation containing sucrose only contained essentially amorphous (noncrystalline) material, whereas the freeze-dried formulation containing glycine only was mostly crystalline material.

In a complex powder mixture of freeze-dried formulation Lead 1, about 70% to 75% of materials were crystalline (Table 4). The overlay of all x-ray powder diffractograms indicates that the pattern of powder of three batches of formulation Lead 1 is the same as that of the glycine-only formulation. No additional peaks were observed for formulation Lead 1. The data suggest that almost all glycine in formulation Lead 1 was crystalline material, but sucrose remained as an amorphous glass matrix stabilizing the subunit vaccine.

**Table 4:** The Ratio of Crystalline to Noncrystalline Structure Based on X-ray Diffraction.

Formulation	Crystalline (%)	Noncrystalline (%)	Model Error
Lead 1 (Batch 1)	75.3	24.7	5.75
Lead 1 with 1% sucrose only	0	100.0	NA
Lead 1 with 3% glycine only	100.0	0	NA
Lead 1 (Batch 2)	70.4	29.6	9.96
Lead 1 (Batch 3)	72.1	27.9	11.90

## Discussion and Conclusion

The active antigen of the H1N1 influenza subunit vaccine that we evaluated consists predominantly of HA and neuraminidase (NA). Similar to other protein molecules, these antigens have limited stability in liquid formulations. Relatively small changes in the external variables (e.g., pH, temperature, salt content) can result in chemical modifications (e.g., hydrolysis) and physical degradation—including aggregation, subunit dissociation, denaturation, unfolding coagulation, and precipitation—and thereby the loss of structural integrity and biological potency of the vaccine.

The most commonly used approach for enhancing the stability of biopharmaceuticals such as vaccine and protein is to develop a formulation that can be converted into a stable dry solid and reconstituted easily into a liquid formulation at the time of use. The presentation of a vaccine as a dry solid may eliminate the need for a cold chain for distribution and may facilitate mass administration of the vaccine and stockpiling.

Freeze-drying (lyophilization) is a process that involves freezing a liquid and subsequent drying by the vaporization of ice under reduced pressure, a phase transition known as sublimation. During the process, the proteinaceous vaccine can suffer from freezing stress and dehydration stress, possibly leading to a loss in potency. A wide range of excipients—including salts, sugars, amino acids, surfactants, and polymers—are often added to the formulation to stabilize the vaccine in both liquid and solid states, as well as through the drying process, by their interaction with the proteins, the solvent (e.g., water), the container surface, and other interfaces [13]. Generally, freeze-drying is the preferred drying method for the reasons stated previously. The application of freeze-drying technology to influenza subunit vaccine was previously reported, and the freeze-dried vaccine powder showed stability for at least 26 weeks at room temperature [20].

We first examined the suitability of commonly used excipients, such as carbohydrates, amino acids, polymers, and salts, in six liquid formulations. Glycine dramatically improved the stability of the liquid formulation at elevated temperatures, while the addition of arginine was detrimental. Having glycine in the formulation was desirable because it has been widely used not only as a buffering agent in the liquid formulation but also as a bulking agent during lyophilization [14]. The saccharide excipients we investigated did not dramatically reduce the thermostability of the liquid formulations in our initial screening, although their stabilizing effect on freeze-dried biopharmaceuticals is well known.

On the basis of these results, in the next step we prepared seven formulations with either disaccharide molecule alone, glycine alone, or a combination of disaccharide and glycine and then evaluated their stability during the lyophilization process and under accelerated storage conditions. As expected, the formulations containing disaccharides, either sucrose or trehalose, demonstrated good stability at a temperature of 37°C. At 45°C, the formulation with trehalose was more stable than that with sucrose. We also observed powder collapse of the sucrose-only formulation at 37°C. Originally, we thought this might be due to the higher moisture content in the sucrose formulation than in the trehalose formulation (2.3% vs. 1.3%); however, the sucrose-only formulation in Round 2 contained a similar moisture content but showed stability after 3 months of storage at 48°C. In fact, the quality of formulation powder obtained from Round 2 was much better than that from Round 1, due to an optimized lyophilization process. The combination of sucrose and dextran 40 also provided a stable formulation, since a polymer such as dextran 40 may add elasticity to reduce cake brittleness. Not surprisingly, use of crystalline bulking agents alone (mannitol or glycine) showed a minimally protective effect because they tend to crystallize during freezing and create a large solid-to-liquid interface that represents a surface area for protein absorption, resulting in conformational change and disruption of vaccine [12,13,14].

Interestingly, the addition of 1% sucrose to the formulation containing glycine or mannitol dramatically improved stability. However, the beneficial effect of sucrose was greater in a sucrose–glycine combination than in a sucrose–mannitol combination. The sucrose–glycine combination system was particularly encouraging, since having glycine in the formulation as a bulking agent may facilitate freeze-drying, reduce the drying cycle time, and make the process more efficient.

The disaccharide–glycine combination system was further evaluated in Round 2 with both sucrose and trehalose. Considering the potential risk of pH change during freezing with sodium phosphate buffer, we also investigated an alternative buffer system, including histidine, which would undergo a minimal pH change during freezing. In the 8-week stability study, all nine formulations tested were stable for the entire period when stored at 37°C or 48°C. Finally, the two lead formulations were selected against additional criteria—including excipient cost, minimal moisture content, freeze-drying time, and formulation complexity—and then scaled up for long-term stability evaluation.

Both lead formulations involve a disaccharide–glycine combination: sucrose–glycine and trehalose–glycine for Lead 1 and Lead 2, respectively. The presence of disaccharides is essential for vaccine stabilization. Sugar can not only form an amorphous glassy matrix, protecting the protein during the process, but also increases the shelf life of the final powder product. Three main mechanisms have been described to support the stabilizing effect of disaccharides: (1) the glassy sugar matrix is highly viscous and immobilizes the drug and water molecules, leading to extremely high activation energy for any reaction to occur [24]; (2) sugar molecules form hydrogen bonds directly with the vaccine, similar to the replaced water [25]; and (3) sugar acts as a physical barrier between two biopharmaceutical molecules, preventing aggregation [26].

Including a crystallizing solute such as glycine in the formulation excipients is also important since glycine crystallization will reduce the drying time during lyophilization. Use of a sucrose–glycine-based excipient system for freeze-dried biopharmaceutical products has been reported in the literature [27,28,29]. Using two model proteins, Liu et al. examined the recovery of protein activity after freeze-drying in a sucrose–glycine-based system in which the ratio of two excipients was varied, and they observed a general pattern in which the recovery of activity decreases as the relative concentration of glycine and the level of crystallinity in the freeze-dried powder increases [25]. However, our lead formulation contained a high ratio of glycine to sucrose (3:1), and the XRD pattern of our Lead 1 formulation powder revealed that most of the glycine in the formulation was crystalline material. The consistent level of crystallinity in three batches suggests a reproducible freeze-drying process. Furthermore, despite phase separation, the physical and biochemical stability of the vaccine was maintained, probably because the amount of amorphous sucrose in the formulation was sufficient to maintain the structural integrity of the vaccine antigen by the mechanism discussed previously. This could also be due to the fact that the lead formulation contains 0.05% of the surfactant, polysorbate 80, which is from the bulk vaccine. The surfactant could eliminate the deteriorative effect of glycine crystallization on protein stability during the lyophilization cycle by occupying the freeze-concentrate/solid interface, and it could protect the vaccine antigen from absorption to this interface thus changing the structural conformation. Overall, a disaccharide (sucrose or trehalose)–glycine-based excipient system plus surfactant provides a stable subunit vaccine formulation for freeze-drying.

Our long-term stability study of the two lead formulations was extended to 40 months to collect more data for accurate shelf life projection. Both formulations remained stable at all storage temperatures studied (4°C, 25°C, and 37°C). The shelf life could be calculated based on 172-week data, using linear regression and the slope of the lower 95% confidence interval. Both formulations have more than 6 years of projected shelf life at 25°C and 37°C and more than 50 years at 2–8°C. This greatly exceeded our target shelf life of 3 to 5 years at 4°C, 3 months at 25°C, and 1 month at 37°C. Furthermore, the antigenic properties of freeze-dried vaccine powder of both lead formulations were maintained, as demonstrated by the results of the in vivo immunogenicity study in mice.

Overall, this work clearly shows that a heat-stable H1N1 influenza subunit vaccine in powder can be obtained by the proper choice of formulation excipients and by applying freeze-drying technology.

We have developed a heat-stable influenza H1N1 subunit vaccine in a dry solid format. This heat-stable freeze-dried vaccine could solve the problem associated with cold chain requirements for liquid influenza vaccines; facilitate vaccine transportation, distribution, and storage; and make the vaccine more accessible and less expensive. We have further demonstrated applicability of this formulation technology to a different influenza subunit vaccine, which will be discussed in detail in a future publication. The greatly extended shelf life of this vaccine can reduce the frequency of turnover of influenza vaccine stockpiles and associated costs, and it can effectively support pandemic preparedness in countries around the world.
